# A novel schistosome species hosted by *Planorbella (Helisoma) trivolvis* is the most widespread swimmer's itch-causing parasite in Michigan inland lakes

**DOI:** 10.1017/S0031182022001561

**Published:** 2023-01

**Authors:** D. M. Soper, T. R. Raffel, J. P. Sckrabulis, K. L. Froelich, B. A. McPhail, M. D. Ostrowski, R. L. Reimink, D. Romano, S. P. Rudko, P. C. Hanington

**Affiliations:** 1 Department of Biology, University of Dallas, 1845 E. Northgate Dr, Irving, TX 75062, USA; 2 Department of Biological Sciences, Oakland University, 118 Library Dr, Rochester Hills, MI 48309, USA; 3 St. Joseph High School, 2521 Stadium Dr, St. Joseph, MI 49085, USA; 4 Freshwater Solutions LLC, 137 W 15th St, Holland, MI 49423, USA; 5 School of Public Health, University of Alberta, 357F South Academic Building, Edmonton, Alberta, Canada T6G 1C9; 6 Office of Campus Ministries, Hope College, Holland, MI 49423, USA; 7 Public Health Agency of Canada, Agence de la Santé Publique du Canada, 200 René-Lévesque Blvd., Montréal, Québec, Canada H2Z 1X4

**Keywords:** eDNA, *Planorbella*, qPCR, *Schistosoma*, snail distribution, swimmer's itch, trematodes, *Trichobilharzia*

## Abstract

Cercarial dermatitis (‘swimmer's itch’; SI), characterized by small itchy bumps caused by schistosome parasites of birds and mammals, is a common problem in Michigan. Research on avian schistosomes began nearly 100 years ago in Michigan inland lakes, yet scientists are still uncovering basic biological information including the identification of local snail and parasite species that cause SI. Previous research primarily focused on lakes in the northern half of Michigan's lower peninsula, although SI occurs throughout the state. We surveyed snails and snail-borne trematodes in lakes across Michigan's lower peninsula and used quantitative polymerase chain reaction analysis of filtered water samples to identify parasites to the species level, including a recently discovered parasite species that uses the snail *Planorbella (Helisoma) trivolvis* as its intermediate host. Most SI mitigation efforts have focused on a parasite species hosted by the snail *Lymnaea catescopium* ( = *Stagnicola emarginata*); however, lymnaeid snails and their associated schistosome species were largely restricted to northern lakes. In contrast, *P. trivolvis* and its associated parasite species were common in both northern and southern Michigan lakes. A third schistosome species associated with physid snails was also present at low levels in both northern and southern lakes. These results indicate that the recently discovered parasite species and its planorbid snail intermediate host may be more important drivers of Michigan SI than previously thought, possibly due to increased definitive host abundance in recent decades. These results have potentially important implications for SI mitigation and control efforts.

## Graphical abstract

**Figure:**
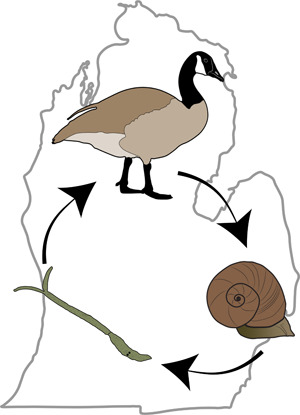

## Introduction

Many swimmers who frequent the waters of Michigan (MI) lakes are familiar with cercarial dermatitis (‘swimmer's itch’), a skin condition caused by snail-borne parasites in the family Schistosomatidae (Muzzall *et al*., 2003; Verbrugge *et al*., 2004; Brant and Loker, 2005; Horák *et al*., 2015; Gordy *et al*., 2018). Swimmer's itch (SI) is generally caused by avian schistosomes in the genus *Trichobilharzia*, which mostly use birds as definitive hosts but sometimes target human swimmers by mistake (Muzzall *et al*., 2003; Brant and Loker, 2009; Horák *et al*., 2015).

The study of avian schistosomes has a long history in MI, including some of the earliest reports of schistosomes in North America (Cort, 1917) and the initial discovery linking these parasites to SI (Cort, 1928). Early reports from the Division of Water Itch Control indicated that ‘from 1939 to 1942 Michigan had the most extensive endemic areas of schistosome dermatitis in the world’ (Cort, 1950), and implicated the single species *Trichobilharzia stagnicolae* as causing ‘most of the swimmer's itch so prevalent on the bathing beaches in northern Michigan’ (Cort *et al*., 1940). Perhaps due in part to this early assessment, studies from 1940 to 2018 focused mostly on the biology of *T. stagnicolae* (hosted by *Lymnaea catescopium* = *Stagnicola emarginata*; Walter, 1969; Burch, 1989; Correa *et al*., 2010) in northern MI inland lakes (McMullen and Brackett, 1948; Keas and Blankespoor, 1997; Muzzall *et al*., 2003; Coady *et al*., 2006; Gordy *et al*., 2018; Rudko *et al*., 2018; McPhail *et al*., 2021). *Lymnaea catescopium* is largely restricted to northern MI (Wall, 1976), which may help explain why SI surveys of snails tended to focus on this region despite reports of SI cases from throughout the state (Clampitt, 1972; Wall, 1976). Less attention was paid to other snail species known to host SI-causing schistosomes (e.g. *Lymnaea stagnalis*, *Physa* spp. and *Gyraulus* spp.; Cort, 1950; Muzzall *et al*., 2003) or to the distribution of snail-borne parasites further south (but see McMullen and Brackett, 1948; Najim, 1956; Guth *et al*., 1979; Kulesa *et al*., 1982; Laman *et al*., 1984*a*; Strohm, 1979) despite increasing reports of SI cases in south MI after 1950 (Clampitt, 1972; Wall, 1976). To our knowledge, only 2 studies to map state-wide gastropod distributions have been conducted in MI, including a survey of SI-producing snails by McMullen and Brackett (1948) and a study that mapped snail observations based on museum specimens (Wall, 1976). Both studies focused on *Lymnaea* and *Physa*.

Of the known host snails present in MI, arguably the most neglected in historical studies is the genus *Planorbella*. We are unaware of a published state-wide distribution map of *Planorbella* in MI, but this genus and in particular *P. trivolvis* have long been considered ‘exceedingly common’ throughout the state (Goodrich, 1932). Almost no historic studies of SI in MI reported screening *Planorbella* snails (e.g. McMullen and Brackett, 1948; Wall, 1976; Muzzall *et al*., 2003; but see Strohm, 1979), despite this being one of the first documented host snails for schistosomes in the state (‘*Cercaria elephantis*’ from Douglas Lake; Cort, 1917). *Cercaria elephantis* did not cause human dermatitis in initial tests (Cort, 1928), but an early study outside MI confirmed that *Planorbella* sp. snails can and do host SI-causing schistosomes (Cort, 1936). Recently, sequencing technology revealed a novel species of SI-inducing schistosome in northern MI lakes, hosted by *Planorbella (Helisoma) trivolvis* and referred to here as Avian Schistosome C (Gordy *et al*., 2018; Rudko *et al*., 2018; McPhail *et al*., 2021).

**Fig. 1.**
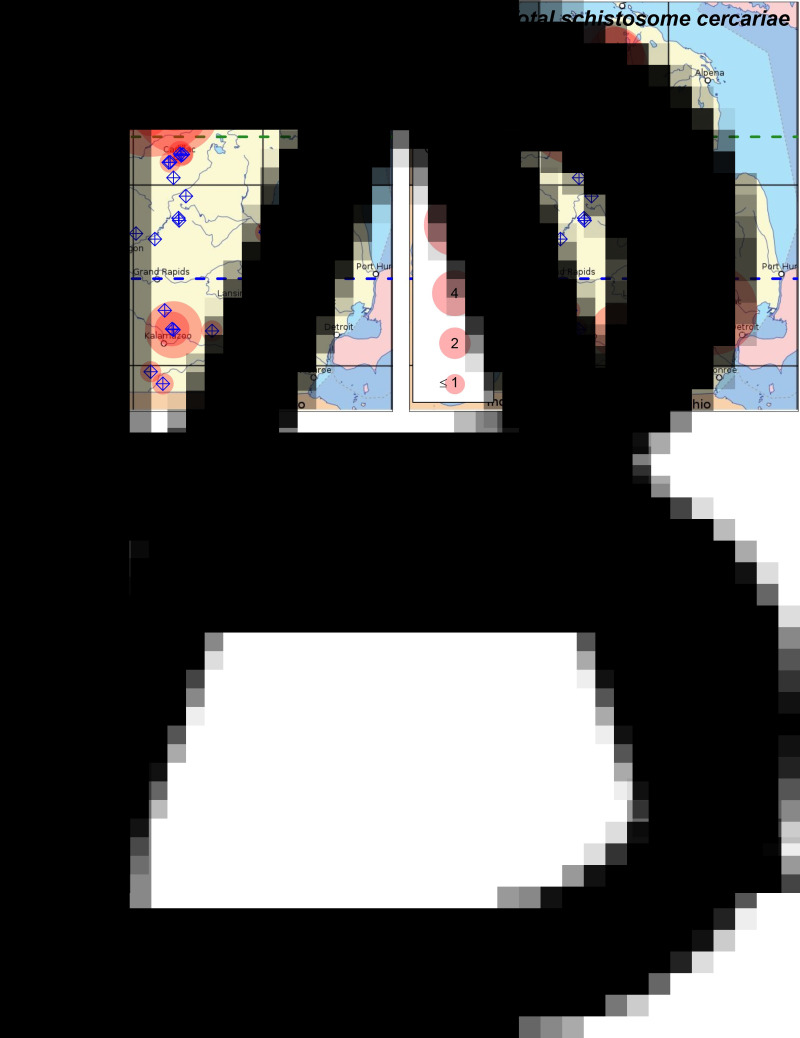
Distributions of (A) known host snails of schistosome cercariae (*Lymnaea*, *Physa*, *Planorbella* or *Gyraulus* spp.) and (B) schistosome cercariae (pan-Schistosoma qPCR assay) in lakes throughout Michigan's lower peninsula. Sampled lakes are indicated with blue target icons. Translucent red circles indicate lakes where host snails or parasites were detected, with circle size indicating (A) mean snails per m^2^ or (B) geometric mean of cercariae per 25 L. Green dashed line indicates boundary between the northern and mid-latitude regions. Blue dashed line indicates boundary between the mid-latitude and southern regions. Lakes where snails were detected in a 30 min search, but were too sparse to be detected in quadrat samples, are indicated with the ‘≤1’ circle size.

We investigated the distribution and abundance of avian schistosomes and their gastropod hosts in inland lakes throughout MI's lower peninsula, including mid-latitude and southern regions (Fig. 1). We collected both live cercariae released from snails and water samples for quantitative polymerase chain reaction (qPCR) analysis, to determine the relative abundance of various schistosome species in the water. Given that SI is observed across the entire state and that Lymnaeid snails are generally restricted to inland lakes in northern MI (Wall, 1976), we hypothesized that other host/parasite species may be responsible for SI in mid-latitude and southern lakes.

## Materials and methods

### Sampling sites and times

We sampled at 128 sites on 43 inland lakes in the lower peninsula of MI (Fig. 1). Lakes and sites were selected to include locations throughout MI's lower peninsula where SI had been documented in past scientific studies (McMullen and Brackett, 1948; Jarcho and van Burkalow, 1952; Kulesa *et al*., 1982; Rudko *et al*., 2018, 2019), news reports (Durham, 2018) or historical state-wide reports of copper sulphate treatment permits and SI control efforts in MI between 1929 and 1976 (Newton and Fetterolf, 1967; Seeburger, 1969; Michigan Bureau of Land and Water Management, 1976). These sites represented a wide range of lake sizes (approximately 30–19 000 acres), shoreline types (e.g. beach *vs* marsh) and levels of human activity. Sampling sites were defined as approximately 15 m × 15 m areas within the littoral zone (<1 m), with shore constraining the shallow border. We conducted all aquatic sampling within the limits of this area. Sampling occurred after infected snails began shedding cercariae, June through August of 2018 and 2019. Some sites were sampled only once while others twice during a single season. Data can be found on Zenodo at https://zenodo.org/record/7221044#.Y07OhHbMLD4.

For analytical purposes, we separated sites into northern (north of Thompsonville), mid-latitude and southern (south of Grand Rapids) regions, roughly corresponding to regions defined by McMullen and Brackett (1948) in their historical state-wide SI survey (see Fig. 1). These regions also roughly correspond to the geographic distribution of level III ecoregions in MI, with forest ecosystems in the north (50: northern lakes and forests; 51: north central hardwood forests), plain ecosystems in the south (55: eastern corn belt plains; 56: southern MI drift plains; 57: Huron/Erie lake plains) and a mix of ecosystems in middle latitudes (Omernik and Griffith, 2014).

### Snail abundance: quadrat sampling

Snail surveys were initially conducted independently by 2 different research groups led by RLR (‘Reimink group’) and TRR (‘Raffel group’); data were later combined for joint analysis and publication. Snail abundance was recorded by visual quadrat sampling at least 1 m^2^ of substrate per site, utilizing either sand-filled round plastic hoops (1 m^2^ inside area; RLR group) or square frames constructed from ½” PVC (0.09 m^2^ inside area; TRR group). These visual quadrat samplers were tossed haphazardly throughout the littoral zone of each site (<1 m depth), and all snails within each quadrat were counted and identified to the genus level using a diving mask (RLR group) or a clear-bottomed view bucket (TRR group). At sites where visual quadrat sampling could not be used to accurately estimate snail densities (e.g. sites with a mucky bottom or a large proportion of vegetation cover), we used a standard pipe sampling method to estimate snail densities. As with visual quadrat surveys, pipe samples were haphazardly distributed throughout the littoral zone (up to 6 total pipe samples per site). For each 0.13 m^2^ pipe sample, we drove a large aluminium pipe through the water column and into the substrate and used a long-handled dip net (4 × 2 mm mesh size) to collect all organisms within the pipe, scraping the bottom substrate to obtain organisms from the benthos. Each pipe sample consisted of a minimum of 10 scoops, after which sampling ended after 5 consecutive scoops yielded no snails. At 38 sites in 28 southern and mid-latitude lakes (TRR group), we also performed an intensive timed search for snails throughout the littoral zone for 30 min, after collecting snail abundance data *via* visual quadrat or pipe samples. Snails were collected for further processing either during quadrat sampling (RLR group) or the timed search (TRR group). All field-collected snails were screened overnight for the production of trematode cercariae (described below). Snails were then preserved in 70% ethanol and brought to the lab for sorting, counting and identification based on gross morphology utilizing the method by Burch (1989).

### Parasite screening and collection

All collected snails were screened for trematode infection by placing snails in fresh spring water (either natural or artificial spring water; Cohen *et al*., 1980) and leaving them in the dark overnight (see Blankespoor and Reimink, 1998). The next morning, the lights were turned on for 1 h to trigger trematode emergence from snails (Kuntz, 1947; McClelland, 1965). After an hour, we examined snails under a stereomicroscope (Leica Microsystems, Wetzlar, Germany S6E) for cercariae in the water. Snails were either screened individually in 6 or 12-well plates, or in groups of 6 in a 250 mL deli cup (depending on the initial number of snails collected at each site). If cercariae were found in a group, then all 6 snails in that deli cup were screened individually over a second night in fresh spring water. Cercaria behaviour was also recorded for representative parasite types using a modified GoPro Hero 3 Black camera fit to the eyepiece of the stereomicroscope. We preserved cercariae from individual snails in 70% ethanol.

### Cercaria staining and mounting

For southern and mid-latitude lakes (*n* = 28; TRR group), a subset of parasites from each snail were preserved in 10% neutral buffered formalin for staining and mounting. These cercariae were stained with Ehrlich's haematoxylin, cleared with methyl salicylate and fixed in Canada balsam according to the procedure described by Dailey (1996). We photographed cercariae under a compound microscope (Leica Microsystems DM1000 LED) fitted with a camera (Leica Microsystems DFC450C). When needed, individual cercariae were photographed at multiple focal lengths to obtain clear images of the entire cercaria, and photographs were then composited together in Adobe Photoshop CC 2022. Cercaria morphotypes were identified based on the key in Schell (1985).

### Avian schistosome cercaria sampling and qPCR cercariometry

**Fig. 2.**
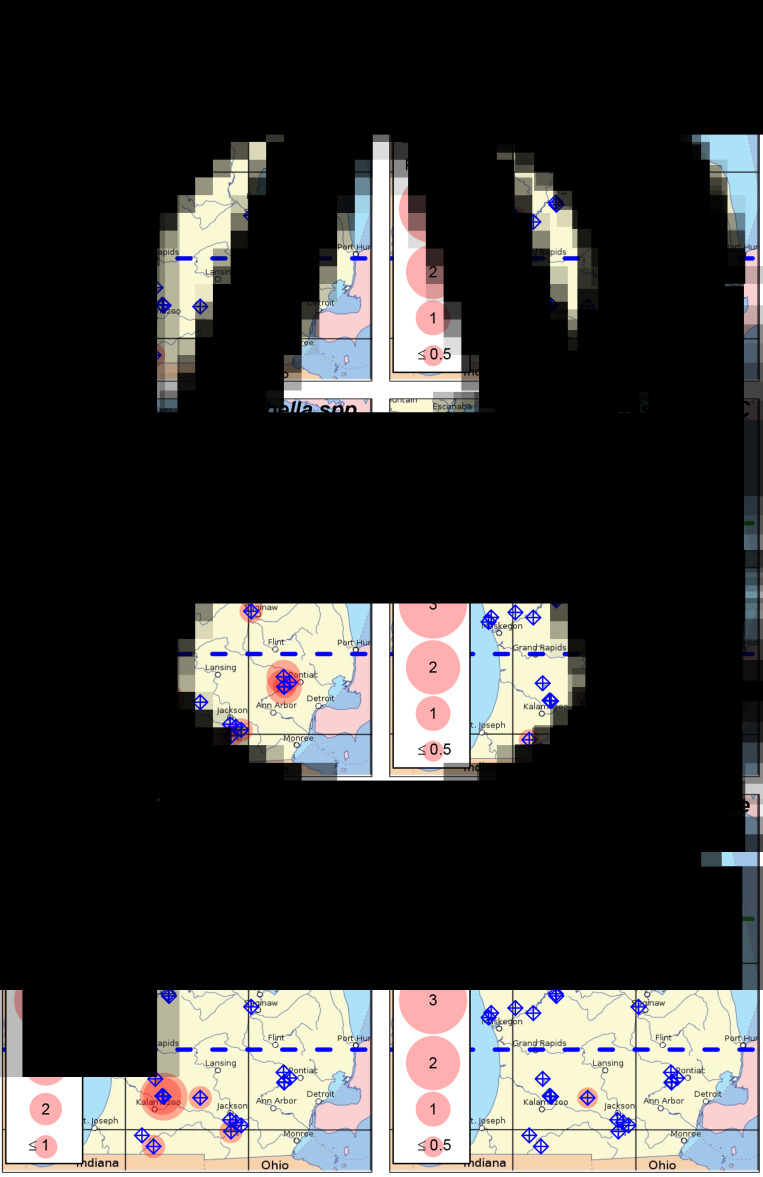
Distribution of known host snails and their associated avian schistosome species in lakes throughout Michigan's lower peninsula. (A, B) *Lymnaea catescopium* ( = ‘*Stagnicola emarginata*’) and *Trichobilharzia stagnicolae*; (C, D) *Planorbella* spp. ( = ‘*Helisoma*’) and Avian Schistosome C; (E, F) *Physa* spp. and *Trichobilharzia physellae*. Sampled lakes are indicated with blue target symbols. Translucent red circles indicate lakes where host snails or parasites were detected, with circle size indicating (A) mean snails per m^2^ or (B) geometric mean of cercariae per 25 L. Green dashed line indicates boundary between the northern and mid-latitude regions. Blue dashed line indicates boundary between the mid-latitude and southern regions. Lakes where snails were detected in a 30 min search, but were too sparse to be detected in quadrat samples, are indicated with the ‘≤1’ circle size.

In both 2018 and 2019, filtered water samples were collected for quantification of avian schistosome DNA. This was a collaborative effort between the RLR and TRR groups starting in 2019. In most but not all cases, filtered water samples were collected during the same site visits as snail surveys. Similar to Rudko *et al*. (2018), each filtered water sample was collected by using a cone-shaped zooplankton net and walking a horseshoe-shaped route starting at the shore and moving approximately 15 m offshore and 15 m along shore (see Figs 1 and 2 for sampling locations). Each step collected approximately 1 L water, and the net was then washed using deionized water followed by a 70% ethanol rinse into a 50 mL falcon tube. Water samples underwent a second filtration through a 0.45 *μ*m filter (Pall) to capture cercariae. DNA was then extracted according to the procedure described in Rudko *et al*. (2020). Briefly, DNA samples were extracted using the Qiagen DNeasy Blood and Tissues kit (Qiagen, USA). qPCR was first conducted using a pan-Schistosoma *18S* rDNA according to Jothikumar *et al*. (2015) and Rudko *et al*. (2018). This initial qPCR assay was performed at the field laboratory in MI using the ChaiBio Open qPCR system (Chai Biotechnologies, USA). For the 2019 surveys, samples positive for avian schistosome DNA were shipped to the University of Alberta and further screened using 5 species-specific qPCR assays targeting *T. stagnicolae*, *Trichobilharzia physellae*, *Trichobilharzia szidati*, *Anserobilharzia brantae* and a putative novel schistosome species Avian Schistosome C *sensu* Gordy *et al*. (2018) and McPhail *et al*. (2021). The species-specific qPCR assays were conducted on the QuantStudio 3 (ThermoFisher Scientific), according to the protocols described and validated by Rudko *et al*. (2019), which include a 1-directional workflow to prevent contamination between pre- and post-amplification spaces. The species-specific assays are highly specific to each target species (Rudko *et al*., 2019; Rudko *et al*., 2022). Species-specific data were converted from gene copy numbers to cercariae per sample by assuming approximately 57,736 copies per cercaria after Rudko *et al*. (2018).

For analytical purposes, we assumed 2019 samples that were negative for the pan-Schistosoma assay would have also been negative for the species-specific assays after negative samples in 2018 also turned up negative in species-specific tests. For 2018 surveys, all samples with sufficient sample volume remaining were assayed with species-specific qPCR, including those that tested negative in the pan-Schistosoma assay. The 2019 qPCR samples included data from Glen Lake (all samples), North and South Lake Leelanau (65 samples each) and Walloon Lake (130 samples) that were previously published by Rudko *et al*. (2022). Five species-specific assay results from Rudko *et al*. (2022) were published as ‘detected but not quantified’, due to being under their specified detection limit. To include these 5 samples in our analysis we assigned them very low values (1 copy number, or approximately 0.000017 cercariae per 25 L; these samples had no noticeable effect on the observed patterns). Data have been deposited in Zenodo prior to publication (https://zenodo.org/record/7221044#.Y07OhHbMLD4).

## Results

### qPCR

We detected avian schistosomes in 30 of the 43 lakes sampled, either with the pan-Schistosoma assay (30 of 43 lakes tested; Fig. 1) or 1 or more species-specific assays (21 of 42 lakes tested; Table 1, Fig. 2). The state-wide distribution of avian schistosome abundance generally matched the distribution of known host snails (Fig. 1), which had high densities in northern lakes, were largely absent from mid-latitude lakes and had moderate to low densities in southern lakes (Table 1, Fig. 1). We detected avian schistosome cercariae in all 15 northern lakes (Figs 1 and 2) and 11 out of 14 southern lakes (Figs 1 and 2). We only detected cercariae in 4 out of 14 mid-latitude lakes, all at low levels (<1 cercariae per 25 L; Figs 1 and 2).

**Table 1.** Detection frequency for avian schistosome species and host snails (genus level) for each of the sampled lakes

Region	Lake	Method	Snail genera				Avian schistosomes		
			*Gyraulus*	*Lymnaea*	*Physa*	*Planorbella*	*T. stagnicolae*	*T. physellae*	Avian schist. C.
North	Bellaire	Q	2/3	3/3^a^	2/3	3/3	16/22	0/22	1/22
	Big Platte	Q	2/12	4/12	9/12	3/12	6/17	3/17	2/17
	Charlevoix	Q	5/6	4/6	6/6	3/6	1/1	1/1	–
	Crooked (Emmet Co.)	Q	1/3	2/3	1/3	3/3	2/9	0/9	4/9
	Elk	Q	1/5	5/5	4/5	4/5	18/29	5/29	12/28
	Glen	–	–	–	–	–	49/130	14/130	27/130
	Intermediate	Q	5/12	5/12	9/12	6/12	7/12	2/12	5/12
	Leelanau – North	Q	4/6	6/6	4/6	4/6	20/67	6/67	11/65
	Leelanau – South	Q	0/6	6/6	5/6	4/6	12/65	12/65	33/65
	Lime	Q	2/2	2/2^a^	1/2	1/2	–	–	–
	Long	Q	3/6	1/6	6/6	6/6	0/41	0/41	4/40
	Pickerel	Q	2/3	3/3^a^	2/3	3/3	2/10	0/10	3/10
	Skegemog	Q	0/4	4/4	4/4	4/4	7/12	0/12	7/11
	Torch	–	–	–	–	–	5/21	0/21	4/21
	Walloon	Q	2/6	6/6^a^	6/6	6/6	50/129	18/129	69/129
Middle	Big	Q,T	0/2	0/2	0/2	0/2	0/2	0/2	0/2
	Bills	Q,T	0/1	0/1	0/1	0/1	0/1	0/1	0/1
	Cadillac	Q,P,T	0/4	0/4	1/4	0/4	0/4	0/4	0/4
	Crooked (Missaukee Co.)	Q,P,T	0/4	0/4	2/4	4/4	0/4	0/4	0/4
	Duck (Muskegon Co.)	Q,T	0/2	0/2	0/2	1/2	0/2	0/2	0/2
	Fremont	Q,T	0/2	0/2	0/2	0/2	1/2^b^	0/2	0/2
	Haithco	Q,T	0/2	0/2	0/2	1/2	0/2	0/2	0/2
	Mecosta	Q,P,T	0/1	0/1	0/1	0/1	0/1	0/1	0/1
	Missaukee	Q,T	0/4	0/4	2/4	3/4	0/4	0/4	1/4
	Mitchell	Q,T	0/2	0/2	0/2	0/2	0/2	0/2	0/2
	Rose	Q,T	0/2	0/2	0/2	0/2	0/2	0/2	0/2
	Round	Q,T	0/2	0/2	0/2	0/2	0/2	0/2	0/2
	Sapphire	Q,T	0/2	0/2	0/2	0/2	0/2	0/2	0/2
	White	Q,P,T	0/1	0/1	0/1	0/1	0/1	0/1	0/1
South	Clark	Q,T	0/2	0/2	0/2	1/2	0/2	0/2	0/2
	Corey	Q,T	0/4	0/4	0/4	4/4	0/4	0/4	1/4
	Devils	Q,P,T	0/4	0/4	1/4	2/4	0/3	0/3	0/3
	Duck (Calhoun Co.)	Q,P,T	0/4	0/4	1/4	0/4	0/4	1/4	4/4^b^
	Evans	Q,T	0/2	0/2	0/2	1/2	0/2	0/2	0/2
	Gull	Q,T	0/2	0/2	1/2	0/2	0/2	0/2	0/2
	Gun	Q,T	0/4	0/4	0/4	0/4	0/4	0/4	0/4
	Klinger	Q,T	0/4	2/4	3/4	3/4	0/4	0/4	0/4
	Lotus	Q,T	0/4	0/4	0/4	2/4	1/4^b^	0/4	1/4
	Maceday	Q,P,T	0/4	0/4	0/4	2/4	0/4	0/4	1/4
	Sylvan	Q,T	0/2	0/2	0/2	0/2	0/2	0/2	1/2^b^
	Upper Straits	Q,P,T	0/2	0/2	0/2	2/2	0/2	0/2	0/2
	Vineyard	Q,T	0/2	0/2	0/2	0/2	0/2	0/2	0/2
	Wintergreen	Q,P,T	0/2	0/2	2/2	2/2	0/2	0/2	0/2

Method indicates which sampling methods were used to survey for snails, as described in the main text: Q (visual quadrat), P (pipe sampling) and T (targeted search). The data are presented as the number of sites where that species was detected/total number of sites sampled at that lake surveyed for snails or conducted species-specific qPCR. Note that there are some lakes where a snail survey was not conducted, species-specific qPCR was not run for 1 or more species of parasite, and that on some lakes we sampled different sites for snails and parasites resulting in mismatched site numbers. Some of the data for Glen Lake, NL Leelanau, SL Leelanau and Walloon Lake were previously published by Rudko *et al*. (2022).

a *Lymnaea stagnalis* was detected at 1 or more sites along with *Lymnaea catescopium* ( = *Stagnicola emarginata*).

b An avian schistosome species was detected at a site, but the corresponding host snail was not detected.

We detected *T. stagnicolae* in 15 of 42 lakes tested, mostly in northern lakes (Fig. 2B, Table 1). The only 2 detections in south or central MI, at Lotus Lake and Fremont Lake, were at very low levels (both <0.1 cercariae per 25 L). This northerly distribution of *T. stagnicolae* matched that of its primary host snail *L. catescopium* ( = ‘*S. emarginata*’), which we only detected at low density in a single southern lake (Klinger Lake; Fig. 2A, Table 1).

We detected *T. physellae* in 9 of 42 lakes tested, including 4 northern lakes and 1 southern lake (Duck Lake in Calhoun County; Fig. 2F, Table 1). All *T. physellae* densities were lower than 1 cercariae per 25 L (Fig. 2F), especially the single detection in a southern lake with an estimate of <0.1 cercariae per 25 L (Duck Lake, Table 1). The state-wide *T. physellae* distribution was generally consistent with that of its *Physa* spp. host snails, which we detected in all 13 northern lakes with snail data, 3 out of 14 mid-latitude lakes and 5 out of 14 southern lakes including Duck Lake (Fig. 2E, Table 1).

We detected Avian Schistosome C in 18 of 41 lakes tested for this species, including all 13 of the tested northern lakes, 1 out of 14 mid-latitude lakes (Lake Missaukee) and 5 out of 14 southern lakes. The state-wide distribution of Avian Schistosome C was largely consistent with the distribution of *Planorbella* snails, which were observed in all 13 northern lakes with snail data, 4 out of 14 mid-latitude lakes and 8 out of 15 southern lakes (Figs 2C and 2D, Table 1). We generally observed higher cercaria densities in northern lakes (Fig. 2D). In southern lakes, we detected relatively high levels in Sylvan Lake (>5 cercariae per 25 L), consistently moderate levels in Duck Lake (Calhoun County; ~0.6 cercariae per 25 L) and lower levels in Corey Lake, Lotus Lake and Maceday Lake (<0.1 cercariae per 25 L).

### State-wide snail distributions

We observed 2 other types of known host snails for avian schistosomes, *L. stagnalis* and *Gyraulus* sp. These were both mostly observed at low densities in northern lakes (Table 1), though it is important to note that *Gyraulus* is a very small benthic snail that would have been easy to miss, especially at muddy southern and mid-latitude sites where they could have passed through the mesh of our pipe sampling nets.

**Fig. 3.**
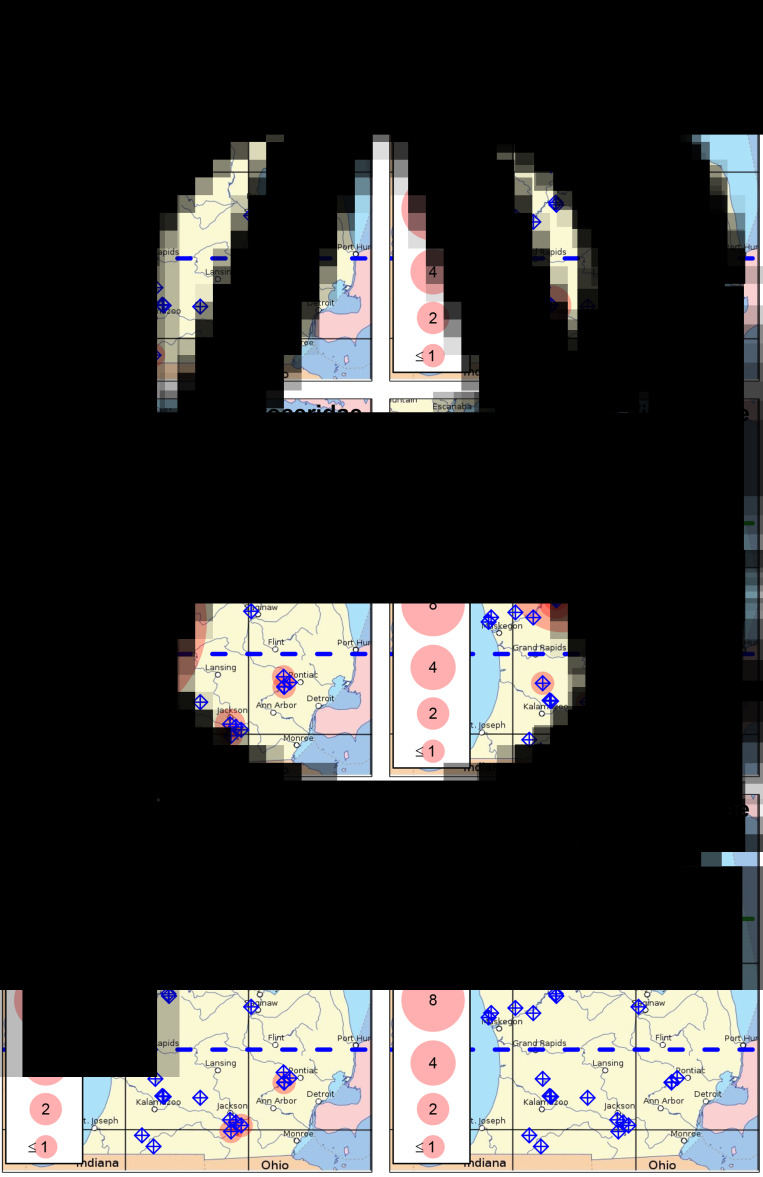
Distribution of snail families in lakes throughout Michigan's lower peninsula, including (A) Lymnaeidae (*Lymnaea catscopium* + *Lymnaea stagnalis*); (B) Planorbidae (*Planorbella* + *Gyraulus*); (C) Pleuroceridae (horn snails); (D) Viviparidae (*Campeloma* + mystery snails); (E) Ampullariidae (apple snails); and (F) Hydrobiidae (mud snails). Physidae was only represented by a single genus (*Physa*), whose distribution is shown in Fig. 2. Sampled lakes are indicated with blue target symbols. Translucent red circles indicate lakes where snails were detected, with circle size indicating mean snails per m^2^. Green dashed line indicates boundary between the northern and mid-latitude regions. Blue dashed line indicates boundary between the mid-latitude and southern regions. Lakes where snails were detected in a 30 min search, but were too sparse to be detected in quadrat samples, are indicated with the ‘≤1’ circle size.

We also observed non-host snails in the families Pleuroceridae (horn snails), Viviparidae (mystery snails and *Camallanus*), Ampullariidae (apple snails) and Hydrobiidae (mud snails; Fig. 3). Pleuroceridae were widespread except in centrally located mid-latitude lakes, with especially high densities in locations near Lake Michigan (Fig. 3C). Viviparidae were also widespread, but in contrast to Pleuroceridae these tended to have higher densities in centrally located mid-latitude lakes (Fig. 3D). Ampullariidae (apple snails) were detected at low densities in 6 southern or mid-latitude lakes (Fig. 3E). Hydrobiidae (mud snails) were detected at low densities in most northern lakes but no southern or mid-latitude lakes (Fig. 3F), though these are also very small snails that may have passed through the mesh of our pipe sampling nets.

## Discussion

The state-wide distribution of avian schistosomes in MI inland lakes generally matched the distribution of known host snails (Fig. 1), with individual parasite species tending to be more common in lakes and regions with higher abundances of their associated intermediate host snails (Fig. 2). However, the correspondence was not perfect, with parasites sometimes being observed in sites where we did not observe their corresponding host snails (or vice versa; Table 1). This result is most likely due to the host snails being outside of our shallow-water sampling areas at these sites, rather than the true absence of intermediate hosts from locations where parasites were detected but not their host snails. Consistent with prior studies, avian schistosome abundances were particularly high in northern MI lakes, especially for *T. stagnicolae* whose lymnaeid intermediate hosts were concentrated in the northern third of the state (Fig. 2). We detected little to no *T. stagnicolae* in southern or mid-latitude lakes, where lymnaeid snails were correspondingly rare (Fig. 2). Even in Klinger Lake, where *L. catescopium* snails were detected during a timed search (Table 1), the density of lymnaeid snails was low enough to escape detection in quadrat samples. Also consistent with prior studies, we detected physid snails and *T. physellae* in both northern and southern MI lakes, though abundances were generally low relative to other species of avian schistosomes and their intermediate host snails.

**Fig. 4.**
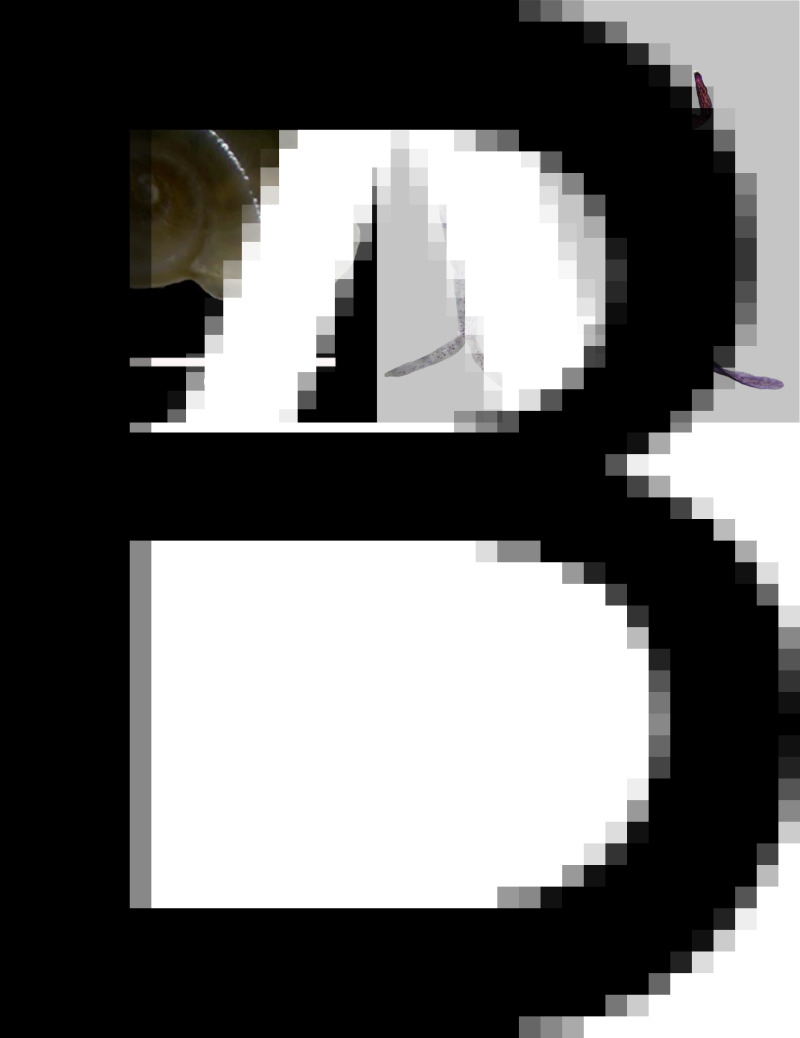
*Planorbella* sp. snail from Lake Missaukee (A) that released brevifurcate-pharyngeate cercaria (B, stained with Ehrlich's haematoxylin). In panel B, the tail of the left cercaria detached during staining; the right cercaria is an intact specimen but was dried out and not mounted flat.

The newly discovered avian schistosome hosted by *Planorbella trivolvis*, first documented and described in Gordy *et al*. (2018) and McPhail *et al*. (2021) and referred to here as ‘Avian Schistosome C’ (Fig. 4), had moderate-to-high cercaria abundances throughout northern and southern MI (Fig. 2). In northern lakes, this species was comparable in abundance to *T. stagnicolae* (Fig. 2). In southern lakes, Avian Schistosome C was the most widespread and abundant avian schistosome species, corresponding with the broad distribution of its *Planorbella* sp. host snails. This result suggests that the new species might be responsible for a large fraction of SI cases throughout MI, especially in the densely populated southern part of the state where *T. stagnicolae* is generally absent. However, much remains unknown about the distribution and potential impact of this parasite species. MI contains thousands of inland lakes (Breck, 2004), and we only sampled a small fraction, especially in the southern region. Furthermore, initial tests seem to indicate that Avian Schistosome C may only cause dermatitis in a small fraction of human volunteers (1 out of 4 volunteers tested; McPhail *et al*., 2021). It is unclear if this is because Avian Schistosome C failed to penetrate the skin of most volunteers or failed to induce a hypersensitivity response following skin penetration (McPhail *et al*., 2021). Some species of avian schistosome have been shown to cause significant tissue damage following skin penetration in mammalian hosts, whether or not the parasite causes dermatitis (Kolářová, 2007; Kolářová *et al*., 2013). *Trichobilharzia regenti* is known to migrate to the central nervous system within mouse hosts, although no data to date exist on the migration of avian schistosomes within human hosts (Horák *et al*., 2015). Taken together, if the lower SI incidence in human trials is due to lower immune reactivity to these parasites, rather than a lower rate of skin penetration, then it will be important to determine whether and how this parasite affects human hosts following skin penetration.

**Fig. 5.**
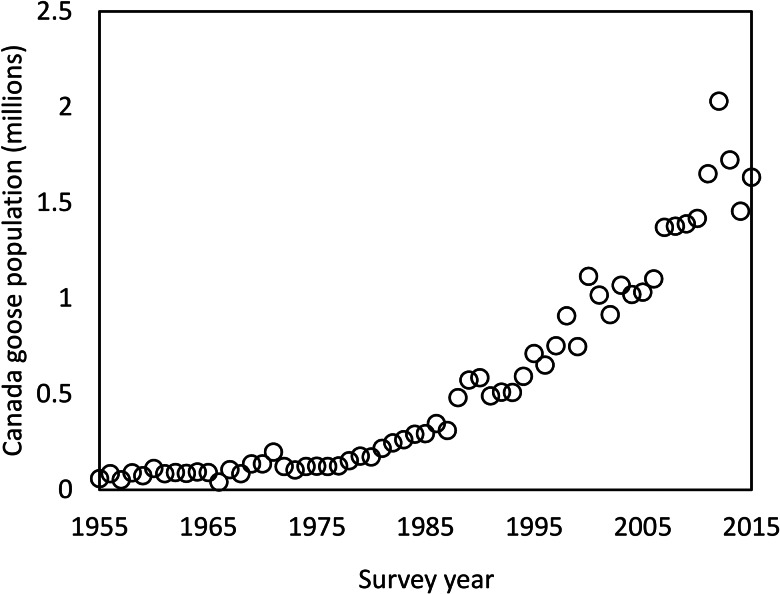
Canada goose population estimate for 1955–2015 in the Midwest-Plains region (Strata 26–50). Data obtained from the annual Waterfowl Breeding Population and Habitat Survey conducted by the Division of Migratory Bird Management of the U.S. Fish & Wildlife Service (U.S. Fish & Wildlife, 2017).

It is surprising that one of the most widespread and abundant avian schistosomes in MI could have gone undetected for so long, despite over a century of avian schistosome research in this state (McPhail *et al*., 2021). Historically, research has been focused on the northern region of MI where *T. stagnicolae* is abundant, and more focus has been placed on that species and its lymnaeid host snail. One other possibility that warrants further investigation is that Avian Schistosome C may be synonymous with one of the schistosome species described in the early 20th century, such as *C. elephantis* which were produced by *Planorbella* in northern MI (Cort, 1917). This parasite failed to produce dermatitis in initial experiments with human volunteers (Cort, 1928) and was largely ignored in subsequent studies (Cort, 1950). However, Avian Schistosome C only caused dermatitis in 1 out of 4 human volunteers (McPhail *et al*., 2021), suggesting that the failure to detect dermatitis in *C. elephantis* by Cort (1928) might have been due to a small sample size and random chance rather than its inability to cause dermatitis. Another possibility is that this parasite recently emerged in MI, either imported from out of state or increasing in abundance from an initially small population. Such an emergence could have been driven by the dramatic increase in its apparent definitive host, Canada geese (*Branta canadensis*; US Fish and Wildlife, 2017; McPhail *et al*., 2021), over the past several decades (Fig. 5). The fact that Canadian geese are a host to Avian Schistosome C may be one reason why merganser (avian host to *T. stagnicolae*) removal has not been effective at eliminating SI from some lakes (Rudko *et al*., 2022). Importation from out of state is plausible due to the continent-wide distribution of the host snail *P. trivolvis* (Martin *et al*., 2020) and the known presence of Avian Schistosome C in Alberta, Canada (Gordy *et al*., 2018; McPhail *et al*., 2021). Further morphological investigation of Avian Schistosome C will be necessary to determine its potential identity with *C. elephantis* (Cort, 1917) or *Cercaria tuckerensis*, a dermatitis-causing schistosome from *Planorbella* snails in Washington State that was described in 1927 (Miller, 1927; Cort, 1936).

In some southern MI lakes, we detected *T. physellae* or Avian Schistosome C but not their associated host species (Fig. 2, Table 1). In other cases, we detected a known host snail species but not its associated schistosome species (Fig. 2, Table 1). This may be due to lower sampling effort in our surveys of southern lakes, since we sampled northern lakes more intensively than southern and mid-latitude lakes in terms of the total area of quadrat sampling and number of site visits (Table 1). Expanded sampling efforts in southern MI lakes are needed to help fill this knowledge gap.

There were 4 lakes where we detected a parasite species without detecting its known corresponding snail host: Lotus Lake and Fremont Lake for *T. stagnicolae*, and Sylvan Lake and Duck Lake (Calhoun County) for Avian Schistosome C. Lotus Lake and Fremont Lake are outside the typical northern range of *L. catescopium*, and each of these detections was based on a single sample with very low levels of qPCR amplification, making it plausible that these may have been false positives. In contrast, Sylvan Lake and Duck Lake are both located near lakes with high *Planorbella* densities, and we detected relatively high levels of parasite DNA in both lakes. Due to the high specificity of trematode parasites for their first intermediate host, it seems almost certain that *Planorbella* sp. are present in these 2 lakes. Avian schistosome cercariae can move long distances in water currents following release from their snail hosts, so the cercariae detected in these lakes may have come from outside of our snail sampling areas (Sckrabulis *et al*., 2020). This highlights the potential utility of species-specific cercaria detection with qPCR as a useful tool for detecting intermediate host snails, especially for snails like *L. catescopium* which prefer deep-water habitats that are difficult to survey (Laman *et al*., 1984*b*).

One unusual result was from Haithco Lake in Saginaw County (Table 1). In 1 of 2 samples, we detected high cercaria levels with the pan-Schistosoma assay (193 cercariae per 25 L) but failed to detect cercariae with any of the 3 species-specific assays. This is a small artificial lake in an urban setting, which we surveyed due to a news report of SI in this lake (Durham, 2018). It is possible that the unusual ecology of this lake, relative to others in this study, has resulted in high abundance of some other avian schistosome species, such as the species that uses *Gyraulus* (Laman *et al*., 1984*a*). We did not detect *Gyraulus* at this lake, but it would have been easy to miss due to its small size and the large amount of submerged vegetation at this sampling site.

Of the regions sampled, mid-latitude lakes had the lowest schistosome levels and correspondingly lowest abundances of host snails (Figs 1 and 2). However, many of these lakes contained large populations of viviparid snails, which tended to be less abundant in northern or southern lakes (Fig. 3D). Many viviparid snails were also infected with trematode parasites, including some with forked-tail vivax cercariae that bear a superficial resemblance to avian schistosomes. However, none of these snails produced the brevifurcate-apharyngeate cercariae that are typical of avian schistosomes (Table 1). Of the remaining snail taxa detected, horn snails were the most widespread and abundant, sometimes dominating the shallow-water snail community in western lakes connected to Lake Michigan (Fig. 3C). In many of the northern lakes, we also detected mud snails at low densities. These were identified to the native genus *Marstonia*, though it is important to note that this genus is physically similar to invasive New Zealand mud snails which have recently been detected in northern MI stream systems (Geist *et al*., 2022).

The results of this study have potentially important implications for managing and preventing schistosome dermatitis in MI inland lakes. Management options that target a single parasite species, such as by removing or treating the primary definitive host (e.g. Blankespoor and Reimink, 1991; Peirce *et al*., 2020), may be ineffective in lakes that harbour other parasite species instead of – or in addition to – the target species (Rudko *et al*., 2022). Management options that focus on preventing contact between swimmers and cercariae, such as floating booms or anti-schistosome skin creams, may be more effective in locations with multiple schistosome species (Salafsky *et al*., 1999).

## Data Availability

Data were deposited in Zenodo upon acceptance of the paper (https://zenodo.org/record/7221044#.Y07OhHbMLD4).
